# Analysis of circadian properties and healthy levels of blue light from smartphones at night

**DOI:** 10.1038/srep11325

**Published:** 2015-06-18

**Authors:** Ji Hye Oh, Heeyeon Yoo, Hoo Keun Park, Young Rag Do

**Affiliations:** 1 Department of Chemistry, Kookmin University, Seoul 136-702, Republic of Korea

## Abstract

This study proposes representative figures of merit for circadian and vision performance for healthy and efficient use of smartphone displays. The recently developed figures of merit for circadian luminous efficacy of radiation (CER) and circadian illuminance (CIL) related to human health and circadian rhythm were measured to compare three kinds of commercial smartphone displays. The CIL values for social network service (SNS) messenger screens from all three displays were higher than 41.3 biolux (blx) in a dark room at night, and the highest CIL value reached 50.9 blx. These CIL values corresponded to melatonin suppression values (MSVs) of 7.3% and 11.4%, respectively. Moreover, smartphone use in a bright room at night had much higher CIL and MSV values (58.7 ~ 105.2 blx and 15.4 ~ 36.1%, respectively). This study also analyzed the nonvisual and visual optical properties of the three smartphone displays while varying the distance between the screen and eye and controlling the brightness setting. Finally, a method to possibly attenuate the unhealthy effects of smartphone displays was proposed and investigated by decreasing the emitting wavelength of blue LEDs in a smartphone LCD backlight and subsequently reducing the circadian effect of the display.

The harmful effects of light at night (LAN) on humans and the environment are now well recognized by the general public. It has long been known that the harmful effects of light on human health may be as important as the healing effects of light on humans^1^,^2^. As previously reported, people exposed to LAN, especially blue light at night, can have increased incidences of a wide range of illnesses including sleep and psychiatric disorders, obesity, diabetes, and several kinds of cancers^3^,^4^. The internal sleep/wake cycle of humans, called the circadian rhythm, adapts to daily changes in exposure to short-wavelength (blue) light in environmental light (natural or artificial) during day and night. It was firmly established in 2007 that melanopsin (intrinsically photosensitive Retinal Ganglion Cells (ipRGC)) photoreceptor which is a subset of retinal ganglion cells in humans) exhibits both dark and light adaptation and regulates through secretion or suppression the level of the hormone melatonin when the level between light and the circadian spectral sensitivity function (C(λ)) match^5^,^6^,^7^,^8^,^9^. The daily production of melatonin can be rapidly suppressed by exposure to blue light, either by short pulses or prolonged illumination, with the short (blue) wavelengths being much more effective than longer wavelengths (red to yellow) in inducing this response^10^. Recently, Falchi *et al.* reported that blue light from artificial light alters natural processes, interferes with melatonin production, and disrupts the circadian rhythm^11^. Consequently, exposure to blue light from artificial light sources, such as a blue-enriched LED lamp, LED backlight for liquid crystal displays (LCDs), and organic light-emitting diodes (OLEDs), late in the evening and at night could be detrimental to human health^12^,^13^,^14^,^15^. In addition, it was also reported that blue light has a greater tendency to affect living organisms by disrupting biological processes that rely on natural cycles of day and night^16^,^17^,^18^. Therefore, light rich in short-wavelength (blue) light should be avoided in the late evening to promote a healthy night-time lighting environment. Managing the light in artificial light sources for optimum health and well-being in a bright night environment is thus critical.

Recently, a few reports have proposed guidelines to optimize the spectral power distribution (SPD) of light from solid-state dichromatic light-emitting diode (LED) lamps for low-illumination outdoor lighting applications in an effort to reduce the amount of light pollution^19^. Furthermore, quite recently, it was reported that four-package white LEDs could be controlled and optimized to achieve a high circadian effect for melatonin suppression/secretion, a high color quality for color perception/reproduction, a high efficiency for energy savings, and tunable figures of merit for the white LED lighting market^20^.

However, harmful blue light at night can be produced from not only various artificial lighting sources, such as LED lamps, fluorescent lamps, and incandescent lamps, but also from video displays, such as OLEDs and LCDs, as people spend an increasing amount of time exposing themselves to blue light emitted from various displays found in TVs, monitors, and smartphones at night. Because of the rapid increase in time and frequency regarding smartphone use, modern humans have been referred to as *homomobilians*^21^. The risk of melatonin suppression at night increases with the increased probability of exposure to smartphone displays because they emit large amounts of blue light. Unfortunately, to date, no reliable reports have analyzed the harmful effects of smartphone displays on circadian properties and human health, although some papers have reported on the risk of blue light from TVs and monitors^22^,^23^,^24^. Moreover, thus far, there have been no concerted efforts to control the spectral power distribution (SPD) of smartphone displays to optimize the non-visual circadian effects at night and vision performance by controlling the operational variables of smartphone displays.

In this study, we analyzed the SPDs of three kinds of smartphone displays, of which two were LCDs and one was OLED, produced by different manufacturers, and their non-visual circadian and visual figures of merit, such as the circadian efficacy of radiation (CER), circadian action factor (CAF), circadian illuminance (CIL), correlated color temperature (CCT), the color gamut, the luminous efficacy of radiation (LER), and the photopic illuminance (PIL)^20^. We also analyzed the circadian and visual optical properties of the three smartphone displays while varying the distance between the screen and eye and controlling the brightness setting of the smartphone displays. Furthermore, we suggested and optimized possible variables to control the SPDs and CILs of smartphone displays by changing the wavelength of the blue LEDs in smartphone LCD backlights to obtain a healthy CIL level in a nighttime environment without significantly disturbing the visual efficiency and color reproducibility of the smartphone displays.

## Experimental

**Figure 1:**
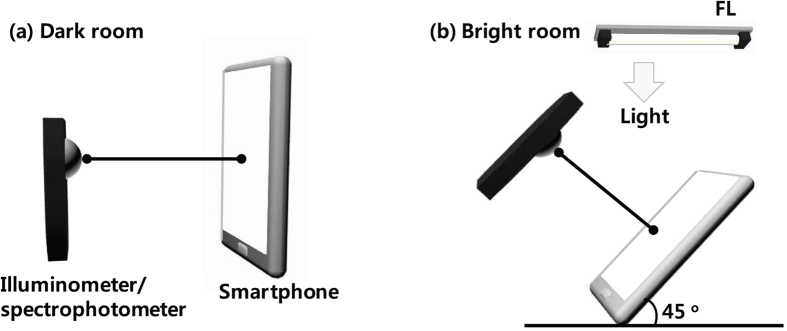
Schematic diagrams of the measurement system in (**a**) a dark room and (**b**) a bright room.

The optical properties for vision performance of the three smartphones (two LCDs (LCD-1: iPhone5, Apple Com.; LCD-2: G3, LG Electronics Inc.) and one AMOLED (Galaxy S5, Samsung Electronics Co. Ltd)) were measured for three different screen modes for viewing web pages (Google, SNS messenger, and a video: public relations (PR) video for Kookmin University) using a spectrophotometer (DARSA-5000, PSI Trading Co.) or illuminometer (TES-1336A light meter, TES instrument) as a function of the smartphone display brightness and the distance between the smartphone display and detector under a 4700 K full-spectrum fluorescent lamp (4700 K FL) (bright room) and under no light (dark room). Figure 1 shows schematic diagrams of the measurement system in dark and bright room environments. As shown in Fig. 1(a), we measured the SPDs and illuminance in the normal direction between the photodetector (or illuminometer) and the three smartphone displays in a dark room environment. In the case of a bright room environment, 4700 K FL was used. The 4700 K FL forms an angle of 45 degrees with the smartphone display (see Fig. 1(b)).

## Results and Discussion

**Figure 2:**
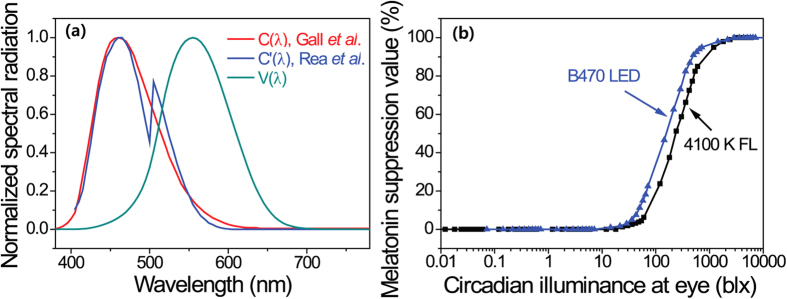
(**a**) The normalized spectra of the vision sensitivity curve (V(λ)), and two kinds of circadian sensitivity curves (C(λ) from Gall *et al.* and C’(λ) from Rea *et al.*). (**b**) The relative melatonin suppression value as a function of circadian illuminance (CIL) from a 4100 K full-spectrum fluorescent lamp (4100 K FL) and a 470 nm blue LED (470 LED).

The effect of the smartphone display SPDs on circadian rhythm, color quality, and vision performance was directly related to the matched levels between the SPDs and the circadian, color, and vision spectral sensitivity curves for each photo-physiological phenomenon considered, respectively. The circadian rhythm, which is the internal sleep/wake cycle of humans, adapts to day and night environments. Several different circadian sensitivity curves (*C’* (*λ*)s) have been reported in previous publications. For example, Branard *et al.*^25^, Tharpan *et al.*^26^, Gall *et al.*^19^,^27^, and Rea *et al.*^28^ have all proposed their own functions. Among them, after comparing two curves from Gall *et al.* (*C* (*λ*)) and Rea *et al.* (*C’* (*λ*)), we selected the circadian spectral function suggested by Gall *et al.* *C* (*λ*)^19^,^27^ to calculate the figures of merit related to human health and the circadian rhythm. As shown in Fig. 2(a), the vision sensitivity curve *V* (*λ*) peaked at about 555 nm, and the color matching functions of the eye consisted of three curves which has been well documented. Otherwise, the circadian sensitivity curve *C* (*λ*)s peaked at about 460 nm, and the shape of *C* (*λ*) was based on data from the action spectrum for melatonin regulation. The circadian rhythm was most sensitive to nearly monochromatic blue light at around 450 ~ 470 nm. Exposure to blue light from any type of smartphone display, including LED-backlight LCDs and active-matrix OLEDs (AMOLEDs), suppressed melatonin. Melatonin is widely known as a biological compound with antioxidant and anti-carcinogenic properties. In addition, controlling the secretion of melatonin is a key factor in the regulation of human health and circadian rhythm.

To analyze the effect of smartphone display SPDs on color, vision, and circadian performance, it is necessary to use figures of merit related to color quality, vision performance, and circadian rhythm (melatonin suppression) for light emitted from these displays. Table 1 summarizes the definitions of all possible and important figures of merit that explain the optical and circadian properties of the smartphone displays. The circadian efficacy of radiation (CER) and the circadian luminous efficacy (CLE) were introduced in previous studies to explain how non-visually bright radiation of the emission spectrum is perceived by the circadian eye system and how brightly non-visual light is emitted from artificial light sources^20^. Similar to the definition of photopic illuminance (PIL), the circadian illuminance (CIL) can also be defined as a measure of how much an incident light illuminates a surface, with the wavelength-weighted by the circadian spectral luminous efficiency function (*C* (*λ*)) to correlate with human circadian perception. In addition, the most important physical value for evaluating the unhealthy effect of displays is the CIL threshold level of blue light which induces melatonin suppression. At night, if we use smartphone displays that are darker than that of the CIL threshold of light which activates circadian systems, the unhealthy effect of smartphone displays can be minimized. However, in the past, there were two conflicting studies on the CIL threshold of light activating melatonin suppression. One report claimed that typical office PIL levels (~ 500 lx) from fluorescent lights were ineffective in their ability to suppress melatonin^29^, whereas another publication reported that low light levels of PIL (~ 3.5 lx) can affect circadian systems^30^. These directly opposing results are because of the irrelevant measurement dimensions and measurement systems of PIL and the different measurement orientations. It is believed that the figures of merit and illuminance values that are ideal for vision are quite different from those that are maximally effective for the circadian system. It is necessary to define meaningful figures of merit related to the non-visual circadian performance of smartphone displays as well as the CIL to measure the total circadian luminous flux incident on a surface per unit area defined in Table 1.

If the CIL is correctly measured for a non-visual point by minimizing both environmental differences (e.g., the distance, orientation, and the presence of reflected objects) and individual differences (e.g., the physical structures of the nose and eyes)^31^, the minimum CIL required to activate circadian perception can subsequently be determined by a series of further medical or biological experiments that assess the simple integrated amount of circadian light flux reaching the ganglion cells of the retina in human eyes. Therefore, complete quantitative control of both the short-wavelength portion of white light and the integrated amount of blue light from any smartphone display is a necessary first step in discussing the potential impact of artificial smartphone displays on human health and well-being. For this reason, it can be again stated that the control of individual smartphone displays and the standardization of the CIL threshold as meaningful data are critical factors in reducing the circadian hazard and optimizing the figures of merit for smartphone displays in terms of both vision performance and circadian effect.

As reported in our previous study^20^, if the threshold levels for the circadian system are measured on a CIL scale, nearly all of the relation curves between the relative melatonin suppression values and CIL values can fall on a single curve from any kind of lighting source, such as artificial lighting sources and smartphone displays. Figure 2(b) shows relative melatonin suppression curves as a function of the calculated CIL from different lighting sources which included a 4100 K full-spectrum fluorescent lamp and a 470 nm blue LED^29^,^32^. Although a single curve was not obtained from the CIL scale, this indicates that the threshold levels of CIL to activate melatonin suppression have similar values regardless of the types of displays or lamps. Here, we can use the relative melatonin suppression curve as a function of the CIL from a 470 nm blue LED as a standard curve to analyze the health effect of individual displays at nighttime because the curve shape from a 470 nm blue LED is well matched with the *C* (*λ*)^32^. As shown in the standard curve in Fig. 2(b), melatonin suppression did not occur below 15 blx (MSV < 1.0%) in any of the displays, and 10% of the melatonin secretion was suppressed at a CIL value above 47 blx from any of the displays or lighting sources.

**Figure 3:**
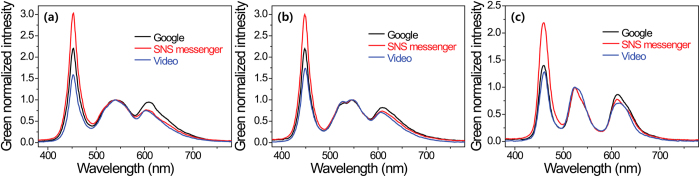
The measured and green normalized spectrum power distributions (SPDs) of three smartphone displays with three different screen modes for viewing web pages (Google, SNS messenger, and a video) (**a**) LCD-1, (**b**) LCD-2 and (**c**) AMOLED.

Figure 3(a–c) show the measured SPDs of the three smartphone displays, which included two LCDs and one AMOLED, with three different screen modes for viewing web pages, represented by Google, SNS messenger, and a video (public relation (PR) video for Kookmin University). These SPDs were measured at a distance of 20 cm between the display and the detector or eye with 100% screen brightness, which can be achieved using the default setting in a dark room. All the smartphone displays showed that their emission spectra had a strong and narrow-band, spike-like blue spectrum, irrespective of the display technology because of the wide color gamut in the displays. It was impossible to manage the distribution and the intensity of the red, green, and blue emission spectra of the smartphones displaying the web pages used in this study at the default brightness setting for the optimization of circadian rhythm as well as color quality and vision performance.

**Figure 4:**
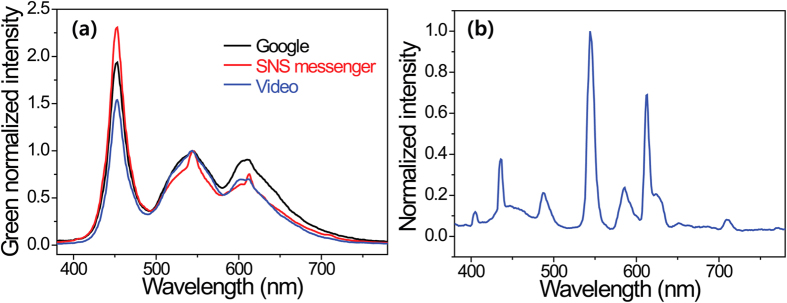
(**a**) The green normalized SPDs of three different screens with the LCD-1 smartphone display in a bright room with 4700 K FL. (**b**) Normalized SPDs of 4700 K FL.

Table 2 presents the calculated and measured circadian and visual figures of merit for the three smartphone displays. All the smartphone displays have similar CER, CAF, and LER values for the same screen modes because they had similar CCT values even though the spectrum distributions were slightly different for each display technology and production brand. Table 2 also includes the calculated CIL values from the measured PIL and CAF values for the three displays. The table shows that still images, such as home pages from messenger and portal sites, were visually and circadianly much brighter than that of dynamic images on smartphone displays, such as the PR video for Kookmin University. The CILs for the social network service (SNS) messenger screens for all three displays had values higher than 41.3 blx, and the highest value reached 50.9 blx. These CILs corresponded to MSVs of 7.3% and 11.4%. These MSVs seem to be low, but they suddenly increased to higher values when the smartphones were used in a bright room (380 lx and 247 blx for a 4700 K fluorescent lamp) at night. We selected the LCD-1 smartphone for another experiment in a bright room. Each smartphone screen has different SPDs in a bright room. As shown in Fig. 4(a), a slightly different spectrum was obtained from the combined spectra of smartphone LCD-1 with a different screen and the room lamp (see Fig. 4(b), 4700 K fluorescent lamp). The reflected light from a turned-off smartphone at a distance of 20 cm had a PIL of 58.3 lx of and a CIL of 37.9 blx. If a smartphone is used in a bright room, the combined light of the smartphone display and the room lighting aggravates the circadian environment around the human user. When we measured the PILs and calculated the CILs of the smartphone LCD-1 display with different screen modes in a bright room, the CILs and MSVs of the smartphone ranged between 58.7 and 105.2 blx and 15.4 and 36.1%, respectively (See Table 2). Compared to the use of a smartphone in a dark room, its use in a bright room has a strong negative impact on the natural cycle of melatonin secretion/suppression in the daytime and on human health by significantly reducing melatonin secretion at night.

**Figure 5:**
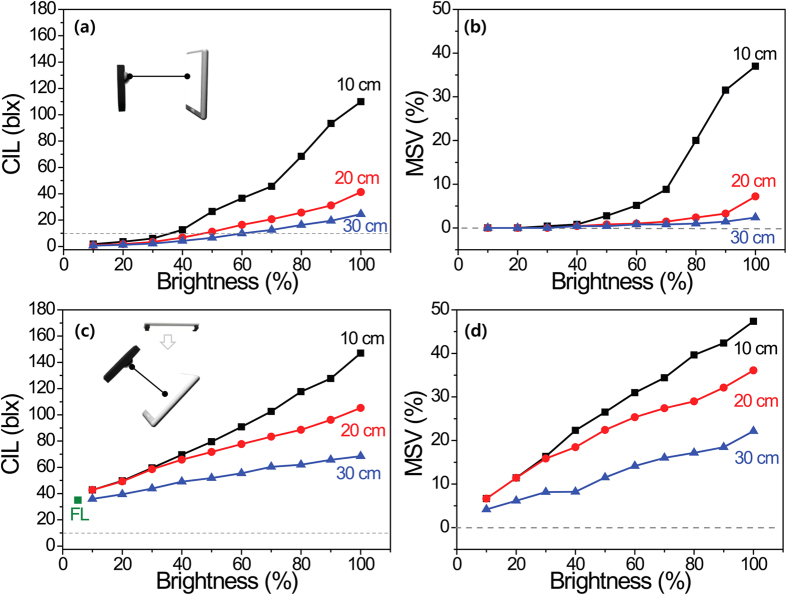
The calculated CILs and MSVs for the SNS messenger screen mode as a function of the display brightness and the distance between the spectrophotometer (or illuminometer) and the smartphone display. (**a**) CILs and (**b**) MSVs; in a dark room. (**c**) CILs and (**d**) MSVs; in a bright room with 4700 K FL.

To study the effect of variations in smartphone use and settings in which users can adjust the circadian properties, smartphone LCD-1 was again used as the standard device for further measurements. Figure 5(a,b) show the calculated CILs and MSVs of a SNS messenger screen with both being functions of the distance between the display and the detector, and the brightness setting of the smartphone display in a dark room. The CILs and MSVs decreased simultaneously with increased distance and a decreased brightness setting, as expected. If we use a smartphone at distances greater than 30 cm with a brightness setting of 100% or a brightness lower than 80% of the maximum value at a distance of 20 cm in a dark room, the MSV falls below about ~2.3%. However, people typically use smartphones with a distance of about 20 cm from the eye to the smartphone with the brightness set as high as possible when using SNS messengers. Therefore, the use of smartphones at night can still have a negative impact on human in terms of circadian rhythm. Figure 5(c,d) show the calculated CILs and MSVs of the SNS messenger screen at a distance of 20 cm in a bright room (380 lx, 4700 K fluorescent room) as a function of the smartphone brightness setting. The results show that the negative effect on melatonin secretion cannot be removed by decreasing the brightness setting of smartphone LCD-1 if the user is typing and reading messages in a bright room.

**Figure 6:**
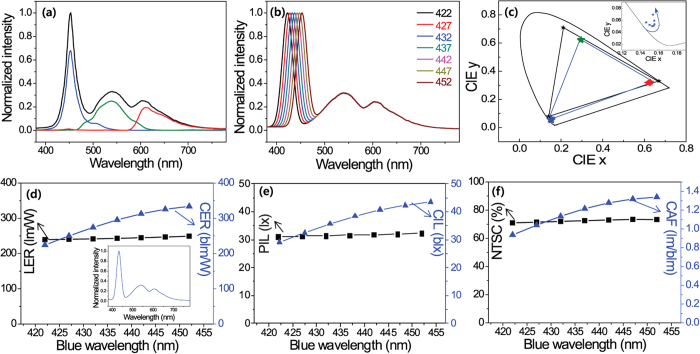
(**a**) The white SPDs of the LCD-1 smartphone display and calculated emission spectra through red, green, and blue color filters. (**b**) Calculated emission spectra by changing the blue peak wavelength from 452 nm to 422 nm. (**c**) The 1931 CIE color coordinates of the red, green, and blue color filtered emissions of the white spectrum from the LCD-1 smartphone with the SNS messenger screen mode (inset: enlarged 1931 CIE color coordinates in the blue region). The figures of merit for the calculated spectra by changing the blue peak wavelength (**d**) LER and CER (inset: white SPD with 432 nm blue peak wavelength), (**e**) PIL and CIL and (**f**) NTSC and CAF.

Night Mode’s main goal is to reduce the brightness of smartphone screens harmful to circadian rhythm to a level that is below the threshold at which a smartphone can induce melatonin suppression. Next, we assumed the use of various bluish violet-emitting InGaN LEDs with a wavelength shorter than a 452 nm blue LED in a LCD backlight to study the possibility of achieving displays for smartphones, monitors, and TVs that are healthier in terms of circadian rhythm^33^. Figure 6(a) shows the white spectrum of a LCD smartphone display and the color filtered spectra of the red (R), green (G), and blue (B) emissions. Figure 6(b) shows the emission spectra of a series of blue and bluish-violet LEDs that have peak wavelengths from 452 to 422 nm with a 5 nm interval. Figure 6(c) shows the 1931 Commission Internationale d’Eclairage (CIE) x, y color coordinates of the color filtered RGB emission of the white spectrum from one smartphone LCD and the change in color coordinates for the blue LED from 452 to 422 nm. The inset of the figure shows the detailed changes of the x and y color coordinates of the blue LEDs in the enlarged CIE diagram. Figures 6(d–f) show the changes in LER, CER, PIL, CIL, CAF, and color gamut at a distance of 20 cm with decreasing emitting wavelengths of blue LEDs obtained by calculating all the figures of merits for circadian and vision performance with a change in the blue spectrum in the combined white emission. The figures show that the more violet LEDs have lower circadian CAF and CER values than that of the blue LEDs, but the more violet LEDs have similar LER values over the whole wavelength range of 422 ~ 452 nm. In addition, the color gamut graph shows that the replacement of more violet LEDs slightly decreases the color gamut of the smartphone LCD display. If we select a 432 nm violet-blue LED for retrofitting a 452 nm blue LED for a smartphone LCD backlight, a violet-shifted white spectrum is obtained, and it can be used as a standard SPD to calculate all the figures of merit shown in the inset of Fig. 6(d). As a result of using a 432 nm blue LED in the smartphone LCD backlight, the CER, CAF, and CIL values decreased from 334 blm/W, 1.34, and 43.4 blx to 274 blm/W, 1.13, and 36.6 blx, respectively; however, the LER and color gamut decreased slightly from 249 lm/W and 73.3 to 242 lm/W and 72, respectively. In addition, these findings show that the CER, CAF, and CIL values decreased by about 15 ~ 18%, but the LER and color gamut decreased by about 0.2% and 0.3%, respectively. For these reasons, it can be readily predicted that the fine control of blue LEDs in smartphone LCD backlights has a greater tendency to affect the circadian performance of smartphones and the circadian rhythm of users but has a lesser tendency to affect the visual performance and the color reproducibility of smartphone LCD displays.

## Conclusions

The figures of merit that are widely used for smartphone displays are the luminous efficacy of radiation (LER), photopic illuminance (PIL) and the luminous efficacy (LE) for vision performance as well as the color gamut for color quality. However, these properties are insufficient when seeking to represent all performances required when analyzing the health effects of individual smartphone displays for use at nighttime. In this study, the previously reported possible figures of merit were explained describing the circadian system in addition to advanced color quality and vision performance. As mentioned above, the circadian luminous efficacy of radiation (CER), the circadian illuminance (CIL), and the circadian action factor (CAF) were calculated and added to the conventional figures of merit for displays to assess the health quality of three commercial smartphone displays (two LCDs and one AMOLED) from different manufacturers. The CIL values for the SNS messenger screens for all three displays were higher than 41.3 blx in a dark room at night, and the highest value reached 50.9 blx. These CILs of the smartphone displays corresponded to MSVs of 7.3% and 11.4%. This CIL level for smartphone displays can significantly decrease melatonin secretion and thereby affect human health and circadian rhythm. If we use smartphones at the proper distance and brightness setting in a dark room, the MSV can drop below ~1%. However, the use of a smartphone in a bright room at night significantly increases the CIL and MSV values ranging from 58.7 to 105.2 blx and from 15.4 to 36.1%, respectively. If people use smartphones in a bright room at night, it will be a little difficult to decrease the blue effect of smartphones on human health by varying smartphone variables that users can adjust. From the results of tuning the emitting wavelength of blue LEDs in a smartphone LCD backlight, fine control of blue light in smartphone displays can have a greater impact on reducing the unhealthy effect of blue light from smartphone displays at night. Thus, combating the unhealthy effect of blue light from smartphone displays at night does not simply mean turning off the smartphone, but rather using well designed SPDs and the proper intensity of smartphone displays to see better where and when required while protecting people’s health and circadian rhythm.

## Additional Information

**How to cite this article**: Oh, J. H. *et al.* Analysis of circadian properties and healthy levels of blue light from smartphones at night. *Sci. Rep.* **5**, 11325; doi: 10.1038/srep11325 (2015).

## Figures and Tables

**Table 1:** The figures of merit for the optical and circadian properties of displays.

Quantity		Symbol
Color quality	Color coordinates (CIEx, CIEy)	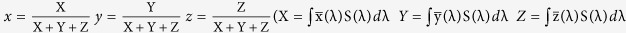
	Correlated color temperature (CCTs, K)	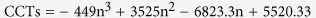
	Color gamut	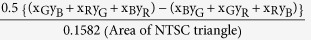 x100 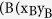 ), 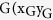 ) 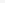 , 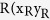 ))
Vision performance	Luminous efficacy of radiation (LER, lm/W)	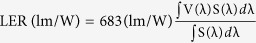
	Luminous efficacy (LE, lm/W)	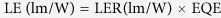
	Photopic Illuminance (PIL, lx)	PIL (lx, lm/m^2^) 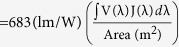
Circadian performance(melatonin suppression)	Circadian luminous efficacy of radiation (CER, blm/W)	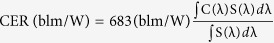
	Circadian action factor (CAF, blm/lm)	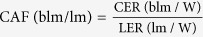
	Circadian Illuminance (CIL, blx)	CIL (blx, blm/m^2^) = PIL (lx, lm/m^2^) x CAF (blm/lm)

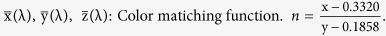
 NTSC color coordinates B(0.14, 0.08), G(0.21, 0.71), R(0.67, 0.33). EQE = External quantum efficiency. J(λ) = Spectral power distribution of the radiation (power per unit wavelength), in watts per meter.

**Table 2:** Calculated and measured circadian and visual figures of merit for the three smartphone displays with different screen modes for viewing web pages (Google, SNS messenger, and a video) in a dark room and a bright room.

		CCT	LER	CER	CAF	PIL	CIL	MSV(470 LED)
		K	lm/W	blm/W	lm/blm	lx	blx	%
LCD-1(Dark room)	Google	6584	281	268	0.95	48.7	46.3	9.0
	SNS messenger	11944	256	318	1.24	33.3	41.3	7.3
	Video	6176	314	258	0.82	25.4	20.9	1.4
LCD-2(Dark room)	Google	7048	276	263	0.95	51.6	49.0	11.1
	SNS messenger	11944	255	307	1.20	42.4	50.9	11.4
	Video	7000	310	268	0.86	25.9	22.4	1.9
AMOLED(Dark room)	Google	6375	278	270	0.97	58.3	56.6	15.0
	SNS messenger	13036	244	334	1.37	35.3	48.4	10.8
	Video	6292	305	279	0.91	27.3	24.9	2.4
LCD-1(Bright room)	Google	6250	282	258	0.92	103.8	95.2	33.1
	SNS messenger	9546	266	304	1.14	92.0	105.2	36.1
	Video	6375	315	264	0.84	70.1	58.7	15.4
